# Inexactness of Medical Writers

**Published:** 1858

**Authors:** A. S. Hudson

**Affiliations:** Professor of Obstetrics in the Medical Department of Iowa University


### THE IOWA MEDICAL JOURNAL.

J. C. HUGHES, } EDITORS. { WELLS R. MARSH.

VOL. IV. KEOKUK, IOWA, JAN. FEB. & MARCH, 1858. NO. 3.

### ORIGINAL COMMUNICATIONS.

### Inexactness of Medical Writers.

BY A. S. HUDSON, M. D.,

(Professor of Obstetrics in the Medical Department of Iowa University.)

It is the province of Medical Journals to keep the profession informed of the progress of medicine. When the literature of the day fails in this particular, then we are left unadvised, as if there were no medical literature beyond the books that are written once in a lifetime. We are left to the casual advantages derived from individual intercourse, and mere hearsay, or the twilight of tradition.

The journals should not only keep the profession informed of every advancing step in the profession, but to them not only knowledge, but *precise* medical knowledge belongs. Hearsay is defective, and tradition is extravagant, vapid, and illusory. If a piece of knowledge is worth knowing at all, it is worth knowing with suitable exactness, precision being an essential attendant upon its usefulness.

We are led to these reflections from the perusal of an article in the Western Lancet, for April 1857, upon Wutzer's plan and instruments for the radical cure of hernia, by the editor, Dr. Blackman, Professor of Surgery in the Ohio

Medical College. Having made some prominent resolves respecting the management of this lesion, we were interested in any thing relating to the subject, and lost no time in addressing ourselves to the perusal of the article. Not having then seen the instrument, we were disappointed that seeing the contents of the article came so far short of standing as an equivalent to seeing the instrument. From subsequent opportunity we find ourselves satisfactorily enlightened, yet no further than what might have been easily done by those two co-working matter-of-fact instructors, the *pen* and the *graver*.

We find the invaginator to be four and a quarter inches long, and half an inch in diameter. Now the article in the Lancet gives no information respecting the size of the instrument. If it is necessary that the properties of length and breadth should belong to it, it is equally necessary they should be stated. The surgeon who is to be instructed, and who may use it, requires the same positive exactness as Mr. Tieman who may construct it.

Dr. Blackman says: "The compressing plate (in his instructions) is now applied, the protruding needle and screw *near its outer extremity* passing through the oval openings of the plate," &c.; which, if his drawing be correct in representing the protruding needle at one end, and the compressing screw at the other, leaves his description out of joint, and to disagree with the drawing, by speaking of parts that stand in opposite position, as if both the screw and protruding needle dwelt in the warm embrace of but one end. The second screw which gives a proper direction to the compressing plates, is like the secret of making an egg stand on end: you are at no loss as soon as you see it done; in like manner you can easily find the screw in the drawing, when once you see the instrument.

Wutzer let the needle remain transfixing the integuments some days, or until he removed the invaginator. This fact is not mentioned in the Lancet. But it is as necessary for the operator to know as to know that the integuments are impaled at all. However, it is gratifying to believe that the operation upon the case succeeded in its object more happily than the report of it.

